# To be or not to be (a prisoner): Social identification as cure and curse via self‐stigma and social exclusion

**DOI:** 10.1111/bjso.70082

**Published:** 2026-04-12

**Authors:** Teresa Traversa, Aoife‐Marie Foran, Marco Marinucci, Luca Pancani, Paolo Riva, Jolanda Jetten

**Affiliations:** ^1^ Department of Psychology University of Milano‐Bicocca Milano Italy; ^2^ School of Psychology University of Queensland Brisbane Queensland Australia; ^3^ School of Psychology University of East Anglia Norwich UK

**Keywords:** prisoners, social cure, social curse, social identification, well‐being

## Abstract

Research on social identification in marginalized populations has documented both social cure and social curse effects, suggesting that distinct identification dimensions may underlie opposite outcomes. This study integrated the Social Identity Approach to Health with stigma and social exclusion research to explore a dual pathway in which ingroup ties and identity centrality are respectively associated with greater or lower well‐being among prisoners through their links with self‐stigma and perceived social exclusion. A path analysis was conducted with a sample of 160 prisoners. Findings suggest that belonging and connectedness derived from identifying with fellow prisoners (i.e., ingroup ties) are associated with reduced psychological distress via lower perceived exclusion. In a context characterized by disconnection, social identification may provide a form of reconnection that supports well‐being. Conversely, the personal importance attributed to the prisoner identity (i.e., identity centrality) was associated with greater self‐stigma and perceived exclusion, with the latter mediating its negative association with well‐being. Within a highly stigmatized group, the centrality of group identity may amplify feelings of exclusion, undermining well‐being. Overall, the study advances understanding of the dual effects of social identification in marginalized groups and underscores the value of applying established psychosocial frameworks to hard‐to‐reach populations.

## INTRODUCTION

Social identities, parts of an individual's self‐concept derived from membership in specific social groups (Tajfel & Turner, 1979), play a central role in shaping individuals' thoughts, feelings, and behaviours, profoundly influencing how people respond to adversity (Haslam et al., 2009). However, belonging to and identifying with a social group is not always beneficial. While identification can foster connection, meaning, and resilience, reflecting social cure processes, it can also relate to heightened distress when group membership is stigmatized or socially devalued, consistent with social curse processes (Jetten et al., 2017). Nevertheless, even in the case of stigmatized identities, the literature addressing the impact of social identification on well‐being presents some inconsistencies (Mazzoni et al., 2020). On the one hand, and consistent with the Rejection‐Identification Model (Branscombe et al., 1999), research shows that identifying with a discriminated group can buffer the impact of stigma and promote well‐being (Jetten et al., 2001; Koc & Hausmann, 2025; Mazzoni et al., 2020). On the other hand, in line with the Identity Threat Model of Stigma (Major & O'Brien, 2005), other studies indicate that such identification can undermine well‐being (Crabtree et al., 2010; Cruwys & Gunaseelan, 2016), functioning as a social curse (Kellezi et al., 2019). This apparent paradox has been observed across various marginalized populations. For example, among immigrants and homeless individuals, both social cure (Bobowik et al., 2017; Johnstone et al., 2015, 2016) and social curse (Bilewicz et al., 2021, 2022; Marinucci et al., 2023) effects have been observed. The present paper seeks to clarify this paradox by examining whether different dimensions of social identification relate to well‐being through distinct mechanisms. In particular, we consider self‐stigma (reflecting the internalization of public stigma) and perceived social exclusion (a central psychological consequence of marginalization). These processes may help explain why identification is associated with a sense of connection and support in some contexts, yet with greater vulnerability and distress in others. To explore these possibilities, we focus on prisoners, an under‐studied population characterized by chronic social exclusion (Marinucci & Riva, 2024), forced cohabitation with fellow group members (Traversa, Marinucci, Riva, & Pancani, 2025), and pervasive stigma (Shi et al., 2022). This provides a theoretically relevant context in which to observe the co‐presence of social cure and social curse pathways, and to investigate potential explanatory processes.

### Social identities and their impact on well‐being

According to the *Social Identity Theory* (Tajfel & Turner, 1979), social identities develop through processes of self‐categorization into social groups, the evaluation of those groups (ingroups) and others (outgroups), and the perceived importance of group membership to the self (Trepte & Loy, 2017). *Self‐Categorization Theory* (Turner et al., 1987), further proposes that the relevance of a particular social identity, and its interplay with personal identity in shaping feelings and behaviours, depends on situational factors and the specific context that may trigger the salience of that social category.

Integrating Social Identity Theory and Self‐Categorization Theory, the *Social Identity Approach to Health* (Haslam et al., 2009) investigates how social identification, meaning the process through which a group becomes relevant and meaningful to one's self‐concept, influences individuals health, well‐being, and behaviour, particularly in response to adverse life experiences. Within this framework, social identification can shape both the primary appraisal of stressors, by influencing how threatening a situation is perceived to be, and the secondary appraisal, by enhancing perceived coping resources, for instance, enabling access to social support from fellow group members (Haslam et al., 2005). As noted earlier, the outcomes of these processes may be beneficial or detrimental, depending on the context‐specific characteristics of the group and the content of the associated social identity. Indeed, while social identification is often associated with enhanced well‐being, when the group is stigmatized or characterized by dysfunctional norms, it may instead foster maladaptive behaviours and poorer mental health (Cruwys et al., 2014).

#### Social identification as a social cure

Groups that are relevant and meaningful to the self can promote individuals' health and well‐being by providing valuable psychological and social resources such as personal security, meaning, agency, and a sense of belonging (Haslam et al., 2009). More specifically, identification with the ingroup can enhance perceived connectedness with fellow members, foster reciprocal support, and strengthen one's sense of purpose, self‐worth, and collective agency (Jetten et al., 2017). Extensive evidence has shown associations between social identification and both mental and physical health outcomes across clinical, community, and workplace settings, including highly vulnerable populations. For example, substantial research indicates that identifying with social groups can reduce depression in diverse populations, such as heart surgery patients and university students (for a review, Cruwys et al., 2014). Comprehensive meta‐analytic evidence has also shown how workgroup and organizational identification are associated with reduced distress and burnout, as well as improved psychological and physical health among employees https://www.zotero.org/google‐docs/?lIUmUv (for a review, Steffens et al., 2017). Finally, in the prison context, and more specifically among prison officers, social identification with this professional group has been shown to mediate the positive effect of high‐quality relationships with colleagues on psychological and occupational well‐being (Traversa, Marinucci, Tortù, et al., 2025).

Social identification can also buffer against the effects of highly stressful life conditions, such as belonging to a stigmatized group (Jetten et al., 2017). This process is described in the *Rejection‐Identification Model* (Branscombe et al., 1999), which posits that identifying with a minority group can mitigate the negative effects of group‐based discrimination on the well‐being of its members. Specifically, while social rejection can directly harm marginalized individuals' well‐being, it can also make the distinction between the ingroup and the outgroup particularly salient, fostering attachment to and identification with the marginalized group. This identification then enables individuals to access psychological and social resources embedded within the group, such as a positive self‐concept and social support, allowing them to cope more effectively with adversity and ultimately enhancing their well‐being (Branscombe et al., 1999). Extensive research has tested and supported the predictions of the Rejection‐Identification Model across diverse populations. For instance, Jetten et al. (2001) experimentally manipulated group‐based discrimination among individuals with body piercings and found that it increased social identification, which in turn predicted higher collective self‐esteem. Similarly, higher social identification with one's ethnic group mediated the association between group‐based discrimination from the national group and decreased antisocial behaviours among adolescents with a migration background (Mazzoni et al., 2020). Finally, recent evidence within the LGBTQ+ context supports the model by showing a positive association between group‐based discrimination and both well‐being and collective action, through the identification with the lesbian group (Koc & Hausmann, 2025). However, research also suggests that group identification can become a threat to well‐being, suggesting that it may be overly simplistic to assume that identifying with a stigmatized group yields only positive outcomes (Crabtree et al., 2010).

#### Social identification as a social curse

When social identity is threatened, such as through stigmatization or under conditions of extreme adversity like war (Kellezi & Reicher, 2012) or immigration detention (Kellezi et al., 2019), social identification can function as a *social curse* (Jetten et al., 2017). For instance, among individuals experiencing mental health difficulties participating in support groups, group identification was directly associated with lower self‐esteem (Crabtree et al., 2010). Similarly, research focused on people experiencing depression showed that perceived stigma was linked to higher identification as a depressed person, which in turn was related to an amplified, rather than buffered, negative association between stigma and well‐being (Cruwys & Gunaseelan, 2016). Finally, among refugees from the Ukrainian war, while identification with the host society (i.e., the outgroup) was associated with lower post‐traumatic stress and greater post‐traumatic growth, identification with fellow refugees (i.e., the ingroup) was linked only to higher post‐traumatic stress (Skrodzka et al., 2024). In line with the Identity Threat Model of Stigma (Major & O'Brien, 2005), this body of research highlights that social identification may amplify the negative consequences of belonging to a group facing stigmatization or extreme adversity by making the group, and its associated negative connotations, particularly salient and personally significant, thereby shaping individuals' self‐image and undermining their well‐being.

#### The differentiated role of identity centrality and ingroup ties

To account for the inconsistent findings regarding the beneficial versus detrimental effects of social identification among marginalized populations, scholars have proposed that different dimensions of social identity may give rise to distinct trajectories. In particular, research has focused on the differentiated roles of *identity centrality* (the extent to which group membership is salient and relevant for the self), *ingroup ties* (the strength of interpersonal connections with other group members), and *ingroup affect* (positive feelings toward the group), the three dimensions described by Cameron (2004). For the purposes of the present work, identity centrality and ingroup ties are of particular relevance, as they capture, respectively, the self‐categorization into a stigmatized group and the sense of bond and shared identity with other group members. Previous research has shown that the beneficial and detrimental pathways of social identification are often driven by these two dimensions. For instance, while ingroup ties and ingroup affect were associated with lower acculturation stress among migrants, identity centrality has been found to be associated with higher levels (Bilewicz et al., 2021). More broadly, the salience and importance of the group for the self appear to underlie the *social curse* trajectory, meaning the negative consequences of identifying with a stigmatized group for well‐being, as observed among homeless individuals, migrants, people with depression, and those from lower social classes (Bilewicz et al., 2022; Cruwys & Gunaseelan, 2016; Marinucci et al., 2023; Rubin & Stuart, 2018). Conversely, the *social cure* path, meaning the beneficial effects of social identification, seems primarily driven by feelings of connectedness, belonging, and social support (Bilewicz et al., 2022; Kellezi et al., 2019; Rubin & Stuart, 2018). Finally, when both pathways were tested simultaneously, identity centrality and ingroup ties emerged as the key dimensions (Bilewicz et al., 2022). It is important to note that the ingroup ties dimension may overlap with purely interpersonal processes, such as social interactions, social bonding, and support, rather than reflecting a group‐based identification process. However, social cure theory (Jetten et al., 2017) highlights how it is precisely the identification process that unlocks the social resources associated with group membership. Thus, we argue that, especially among marginalized populations, where identifying with the group may be associated with both positive and negative outcomes, the group dimension remains central and warrants specific investigation.

Although not explicitly addressing the centrality and ties dimensions, the qualitative work by Bradshaw and Muldoon (2020) explored the negative and positive identity dynamics of women participating in a group‐based support program for partners of incarcerated men. On the one hand, the participants described strong feelings of social isolation and exclusion due to the strongly stigmatized identity, exacerbated by the reluctance to seek help in order to avoid disclosing their situation. On the other hand, sharing the same experience with others allowed them to feel understood and connected with fellow group members, providing a sense of support and belonging. This work highlights how the same stigmatized group identity can simultaneously be both a threat due to its negative attributes and the potential further isolation stemming from concealment, and a resource in terms of social support and belongingness through the awareness of sharing that experience with others (Bradshaw & Muldoon, 2020).

The existence of different pathways associated with identity centrality and ingroup ties seems also consistent with the themes identified by Kellezi et al. (2019) in their qualitative study of immigration detainees. On the one hand, becoming a detainee was associated with the awareness of the associated social exclusion, discrimination, dehumanization, and a loss of agency and control. On the other hand, the emerging detainee identity was also associated with practical and emotional support, as well as empathy and understanding, which helped group members cope with the adverse experience of incarceration (Kellezi et al., 2019). Consistently, Rubin and Stuart (2018) showed how the psychological centrality of social class identity threatened well‐being, while the aspects of social identification associated with a sense of belonging and connectedness with other members of one's social class promoted it. However, this dual pathway has never been empirically tested in an extreme context of social exclusion such as prisons.

#### The mediating role of self‐stigma and social exclusion

While the literature has partially examined the differentiated roles of various dimensions of social identification in shaping the well‐being of marginalized populations, less attention has been given to the underlying mechanisms driving these distinct trajectories. In the present paper, we aim at exploring how the two opposing pathways linked to different dimensions of social identity can be partially explained by the mediating effects of self‐stigma (also referred to as internalized stigma) and perceived social exclusion, both associated with adverse consequences for marginalized individuals' health. Self‐stigma, which occurs when individuals internalize negative attitudes and stereotypes about themselves (Corrigan & Rao, 2012), has been consistently linked to higher levels of depression across marginalized groups (for a review, see O'Donnell & Foran, 2024). Likewise, prolonged and pervasive social exclusion, defined as the experience of being kept apart from others physically or emotionally (Riva & Eck, 2016), can culminate in a state of resignation, characterized by depression, alienation, feelings of unworthiness and helplessness, for instance among people experiencing homelessness (Marinucci et al., 2023; Williams, 2009). In the present paper, self‐stigma refers to the internalization of the negative evaluation of the prisoner group, while perceived social exclusion refers to the awareness of the physical and psychological separation from the outside world and the consequent relational deprivation.

According to the Identity Threat Model of Stigma (Major & O'Brien, 2005), identifying with a marginalized group can exacerbate the negative consequences of stigmatization, making group identification potentially harmful. In line with Begeny and Huo (2018), we propose that the saliency and personal importance of the prisoner identity (i.e., identity centrality) may be associated with greater internalization of the stigma associated with this group (i.e., self‐stigma). Similarly, as shown by Marinucci et al. (2023), identifying with a socially excluded group can amplify perceptions of rejection and ostracism (i.e., perceived social exclusion), thereby threatening well‐being. Conversely, the sense of belonging and connectedness derived from social identification (i.e., ingroup ties) may serve as a protective factor by reducing self‐stigma and perceived social exclusion. Consistent with this view, previous research has shown that identification with stigmatized groups is associated with greater social support and adaptive coping strategies, such as stigma resistance, which in turn promote well‐being (Crabtree et al., 2010). However, the potential explanatory role of factors such as self‐stigma and perceived social exclusion in the dual relationship between social identification and well‐being has not yet been empirically examined.

### Social identity processes in and beyond the prison context

Prisoners, as criminal offenders, constitute a highly stigmatized population who evoke perceptions of dangerousness, distrust, dehumanization, and social avoidance (Shi et al., 2022). Furthermore, imprisonment represents a pervasive, institutionalized, and legitimized form of social exclusion (Aureli et al., 2020; Traversa, Marinucci, Tortù, et al., 2025) marked by physical separation from society and prior networks, alongside forced cohabitation with other inmates under crowded conditions (Schliehe et al., 2022). These structural and social conditions are likely to shape prisoners' social identity processes. As previous social bonds weaken (Schaefer et al., 2017) and individuals adapt to a closed, highly constrained social environment (Traversa, Marinucci, Riva, & Pancani, 2025), the prisoner group often becomes one of the few accessible sources of social (re)connection and support. At the same time, identifying with the prisoner group means coping with a *spoiled identity*, what Goffman (1963) termed “blemishes of character” referring to traits perceived as morally or behaviorally undesirable.

Existing research on group membership and identification within the prison context remains relatively limited, employing diverse measures and approaches. Kyprianides and Easterbrook (2020a) investigated the social cure properties of a music project in UK prisons, showing through both longitudinal questionnaire and interview data how identification with the music group promoted prisoners' well‐being by satisfying their psychological needs, particularly social support and connectedness. The possibility of building a positive identity alternative to the prisoner one was also identified as a key buffering mechanism against the negative consequences of imprisonment. However, the study employed an overall measure of social identification, without distinguishing between identity centrality and ties, and did not consider identification with the broader prisoner category. Furthermore, Kyprianides and Easterbrook (2020b), through secondary analyses of a large UK prison sample, found that positive interactions with fellow prisoners and belonging to a greater number of prisoner groups (i.e., *multiple group membership*) were associated with higher well‐being. Similarly, Aureli et al. (2020) reported that participation in a prison‐based support group predicted lower levels of resignation through increased perceived social support. Although these studies did not specifically assess the degree of identification with the broader prisoner category, they suggest that a sense of belonging and connectedness with fellow prisoners, usually linked to the ingroup ties dimension, can enhance well‐being.

Conversely, when applying the Rejection‐Identification Model to ex‐prisoners, Kyprianides and colleagues (Kyprianides et al., 2019) found that the stronger identification with the ex‐prisoner group, arising from group‐based discrimination, threatened rather than protected well‐being, with multiple group membership further increasing the social curse effect. Similarly, among current prisoners, higher identification with the prisoner group was associated with lower well‐being (Mosso et al., 2024). These results suggest that identification with the prisoner group can also reflect a different psychological process that, in line with the Identity Threat Model of Stigma (Major & O'Brien, 2005), may heighten awareness of social devaluation and rejection, thereby undermining well‐being.

Imprisonment strongly affects prisoners' families, especially children (Poehlmann‐Tynan & Turney, 2021) and partners (Kotova, 2019). Bradshaw and colleagues (2024) showed that experiencing parental incarceration and its associated stigma can reduce social connection among children of incarcerated parents, acting as a social curse, while maintaining multiple group memberships can buffer its negative consequences and promote well‐being, acting as a social cure. Furthermore, as noted above, the group identity of partners of incarcerated men both represents a threat, due to stigma and concealment, and a resource, in terms of social connectedness (Bradshaw & Muldoon, 2020).

These findings suggest that a dual process may also operate within and beyond prisons, with different dimensions of social identification being associated with opposing outcomes for prisoners', ex‐prisoners', and their families' well‐being. However, the existing literature did not always specifically address the subjective process of social identification (e.g., when investigating multiple group membership or the quality of social interactions), and did not explicitly distinguish between identity centrality and ingroup ties. The present study aims to address this gap by examining two distinct dimensions of the identification process, alongside two potential explanatory variables that remain largely unexplored in this context. Specifically, we hypothesise that ingroup ties, encompassing feelings of social connectedness and belonging, will be linked to better well‐being among prisoners, whereas identity centrality, reflecting the salience and personal importance of the prisoner identity, will likely undermine it. Furthermore, we propose that self‐stigma and perceived social exclusion, both central aspects of the prison experience, may play an explanatory role in these relationships. By exploring these hypotheses, the present study expands the existing literature by simultaneously examining the role of two distinct dimensions of social identification and two processes that may be linked to well‐being in a highly stigmatized, socially excluded, and understudied population, namely, prisoners.

## THE PRESENT STUDY

Social identity processes in marginalized populations, such as prisoners, may show opposing associations with well‐being through distinct mechanisms. First, the social connectedness derived from social identification (i.e., ingroup ties) may help prisoners cope with the profound social exclusion inherent in imprisonment and the stigma associated with the group. At the same time, the subjective importance of group membership (identity centrality) may be associated with higher self‐stigma and perceived social exclusion, with negative consequences on well‐being.

The present study seeks to expand the existing literature by exploring these two potential pathways within a population characterized by chronic exclusion and high levels of stigma, namely prisoners. Specifically, we examine whether different dimensions of social identification, that is, identity centrality and ingroup ties, relate differently to prisoners' well‐being, and whether these relationships are explained by self‐stigma and perceived social exclusion. Based on previous evidence suggesting that close bonds with others in similar conditions can provide social and psychological resources (Crabtree et al., 2010; Kyprianides & Easterbrook, 2020b), we expect that stronger ingroup ties will be associated with lower self‐stigma and perceived social exclusion, and consequently with greater well‐being. Conversely, drawing on research indicating that the salience and importance of social identification (i.e., identity centrality) with stigmatized and socially devalued groups can intensify negative outcomes (Begeny & Huo, 2018; Marinucci et al., 2023), we explore whether greater identity centrality is associated with greater self‐stigma and perceived social exclusion, and in turn with lower well‐being.

It is important to note that the phenomena examined in the present study could also be interpreted within the framework of the Rejection‐Identification Model (Branscombe et al., 1999), which posits that stigma and discrimination influence well‐being through the mediating role of social identification. However, the original model focused on individuals' tendency to attribute negative experiences in daily life to group‐based discrimination, rather than on subjective experiences such as self‐stigma and perceived social exclusion, as considered here. Specifically, we propose that social identification may be associated with well‐being through its links with the internalization of both stigma and feelings of exclusion, whereas the Rejection‐Identification Model (Branscombe et al., 1999) emphasizes the buffering role of identification in the relationship between the exposure to group‐based discrimination and well‐being. Furthermore, prior research has already explored related mechanisms in which perceived discrimination (Begeny & Huo, 2018) and perceived social exclusion (Marinucci et al., 2023) mediated the effect of social identification on well‐being. However, previous empirical studies have less systematically and intensively tested social identification processes in light of the Identity Threat Model of Stigma (Major & O'Brien, 2005), considering social identity as predictors of group and social perception. Nevertheless, for completeness, the predictions of the Rejection‐Identification Model (Branscombe et al., 1999) will also be tested as an alternative model.

All study materials, data, and the analysis script can be consulted at this link: https://osf.io/7a4df/overview?view_only=0666c352bb474732832db7de3976eceb.

## METHOD

### Design and procedure

The present study employs a correlational design, drawing on data from the second wave of a larger three‐wave longitudinal project, with six‐month intervals between each wave. The utilized wave was selected because it included the full set of measures relevant to the present study, which were not available at baseline. Additionally, the number of participants who completed both Wave 2 and Wave 3, which included the variables relevant to the present study, was insufficient to longitudinally test the hypothesised mediation model with adequate statistical power. Accordingly, the current analyses focus on cross‐sectional associations among the variables of interest within the second wave sample. Following approval from the Italian Ministry of Justice and the Ethics Committee of the University of Milano‐Bicocca, data collection was conducted in three correctional facilities in northern Italy. In one facility, recruitment was managed peer‐to‐peer by inmates collaborating with the research team; in the other two, prison staff invited eligible participants. The research team requested the application of two exclusion criteria: established and manifest psychiatric disorders and insufficient comprehension of written Italian. Participants were organized into groups of no more than 15 and gathered in communal prison areas (e.g., libraries or classrooms). Sessions were attended only by the researchers and participants, who were assured that their involvement would have no impact on their prison conditions or future trajectories. In the first wave, the researchers introduced the project, explained the voluntary and anonymous nature of participation, and obtained informed consent covering all three waves. Paper questionnaires were administered following a brief explanation of the study. A total of 276 prisoners participated in the first wave. In the second and third wave the study was briefly reintroduced with the same assurances of confidentiality, with 160 participants completing the second wave and 102 the third. The substantial attrition was primarily due to participants being released or granted prison benefits, such as parole.

### Participants

The sample included 159 male prisoners and one female prisoner. The female participant was excluded because she was housed in a different section of the prison with conditions not comparable to the male sections. Additionally, as a single case, her inclusion would not have permitted a meaningful investigation of the female experience. Two other participants were excluded for not completing the survey, resulting in a final sample size of 157. The respondents' average age, measured at T0 (six months before T1), is 39.8 years (*SD* = 13.05, range = 18–72 years). In terms of educational attainment, the majority had completed middle school (54.8%), followed by high school (26.5%), higher education (10.3%), and primary school (7.1%). Two participants (1.3%) reported having no formal education. In terms of relationship status, 33.3% of participants were married or in a stable relationship. The remaining respondents were single, separated or divorced (66.0%), or widowed (0.7%). Most respondents were Italian (67.2%), while 32.8% were originally from other countries. Additionally, more than half of the sample (53.9%) reported having children.

Participants were detained in three different correctional facilities in Northern Italy: one large prison mainly characterized by medium‐to‐long sentence inmates (*N*_1_ = 75, 48.4%), and two medium‐sized jails characterized by short‐to‐medium sentenced detainees (*N*_2_ = 32, 20.4%; *N*_3_ = 49, 31.2%). Across the three facilities, 112 participants (71.3%) followed a normal medium security regime, while 45 (27.7%) followed an advanced treatment regime. The average sentence length was 9.04 years (*SD* = 6.75, range = 1.4–39 years). Participants had, on average, already spent 4.52 years in prison (*SD* = 4.78, range = 0.5–33 years) and had 4.50 years remaining until release (*SD* = 3.69, range = 0–23 years).

### Measures

The current study is based on the subset of the variables measured in the overall questionnaire that are relevant to the study hypotheses. Responses were provided on a 5‐point Likert scale (ranging from 1 = “Not at all” to 5 = “Extremely”), and the scores correspond to the items' average.

#### Social identification

Participants' social identification with the prisoner group was assessed using six items from Cameron's (2004) Multidimensional Scale of Social Identification, specifically from the *ingroup ties* and *identity centrality* subscales. Three of these items measured ingroup ties (e.g., “I feel strong ties to other prisoners”), and the other three measured identity centrality (e.g., “Being a prisoner is an important aspect of who I am”). A principal component analysis and a parallel analysis on all six items supported a two‐factor solution (*R* ^2^ = .62), consistent with the original multidimensional structure of the scale (Cameron, 2004). The two components corresponded to ingroup ties (*λ*s_range_ = 0.72–0.86, Cronbach's *α* = .75) and identity centrality (*λ*s_range_ = 0.46–0.87, Cronbach's *α* = .60). Mean scores were computed separately for each subscale.

#### Self‐stigma

Self‐stigma was assessed using three adapted items (e.g., “I am ashamed of being a prisoner”) from Mak and Cheung's (2010) Self‐Stigma Scale. A principal component analysis indicated that all items loaded onto a single component (*R* ^2^ = .72, *λ*s_range_ = 0.81–0.91; Cronbach's *α* = .80).

#### Social exclusion

Feelings of social exclusion were assessed using three adapted items (e.g., “I feel rejected”) from Williams (2009). A principal component analysis indicated that all items loaded onto a single component (*R* ^2^ = .82, *λ*s_range_ = 0.88–0.93; Cronbach's *α* = .89).

#### Psychological distress

Psychological distress was conceptualized as *resignation*, which is consistent with the final stage of the response to chronic social exclusion as described by Williams (2009). We adopted the same 12‐item scale as Traversa and colleagues (Traversa, Marinucci, Tortù, et al., 2025), to assess depression (e.g., “I couldn't feel positive emotions”), alienation (e.g., “I felt distant from people”), unworthiness (e.g., “Sometimes I thought I was useless”), and helplessness (e.g., “My future seemed vague and uncertain”). A principal component analysis showed that the 12 items loaded onto a single component (*R* ^2^ = .41, *λ*s_range_ = 0.20–0.84; Cronbach's *α* = .85), although one item exhibited poor fit. After removing this item, a second analysis confirmed a single‐factor solution (*R* ^2^ = .44, *λ*s_range_ = 0.33–0.84; Cronbach's *α* = .87).

### Analytic strategy

To test the hypothesised model we included ingroup ties and centrality as the main predictors, self‐stigma and exclusion as mediators, and psychological distress as the outcome. The multiple mediation model was estimated using path analysis in RStudio (Posit Team, 2024) employing the *lavaan* package (Rosseel, 2012). As the model was saturated, the fit was perfect by definition (*df* = 0) and could not be tested (West et al., 2012). However, AIC and BIC values are reported for transparency. Parameter estimates were obtained using bootstrapping with 5000 resamples. Full‐information maximum likelihood (FIML) estimation was applied to handle missing data (Li & Lomax, 2017). The sample size allowed us to have sufficient power, as, following Kline's (2023) recommendations, which suggest collecting at least 10 cases per estimated parameter, the required sample size was 150. The final sample of 157 participants slightly exceeded this minimum.

Due to the correlational nature of the study, alternative pathways to those outlined in the hypothesised model are possible. Therefore, as previously mentioned, we also tested an alternative model that was more consistent with the Rejection‐Identification Model (Branscombe et al., 1999). In this model, self‐stigma and perceived social exclusion predict psychological distress via the mediation of ingroup ties and identity centrality. Furthermore, to control for the potential intervening effect of the temporal dimension, we ran the same model, this time adding the time already spent in prison as a covariate. However, adding three new parameters would require a sample size of 180, rendering this analysis underpowered and the results to be interpreted with caution. Analysis codes and detailed results for these additional analyses are available in the Additional Analyses file (OSF folder: https://osf.io/7a4df/overview?view_only=0666c352bb474732832db7de3976eceb).

## RESULTS

Following the calculation of descriptive statistics, including means, standard deviations, and bivariate correlations between the variables under investigation (see Table 1), the hypothesised path model was estimated.

**TABLE 1:** Mean (standard deviation) and Pearson correlations of the considered variables.

Variable		*M* (*SD*)	1	2	3	4
1	Ingroup ties	2.53 (0.81)	–			
2	Centrality	2.26 (0.86)	.24*	–		
3	Self‐stigma	2.75 (1.26)	−.04	.22*	–	
4	Perceived social exclusion	1.91 (1.16)	−.12	.24*	.41***	
5	Psychological distress	2.21 (0.83)	−.02	.28**	.43***	.72***

*Note*: *p*‐Values are adjusted for multiple comparisons.

* *p* < .05;

** *p* < .01;

*** *p* < .001.

**FIGURE 1:**
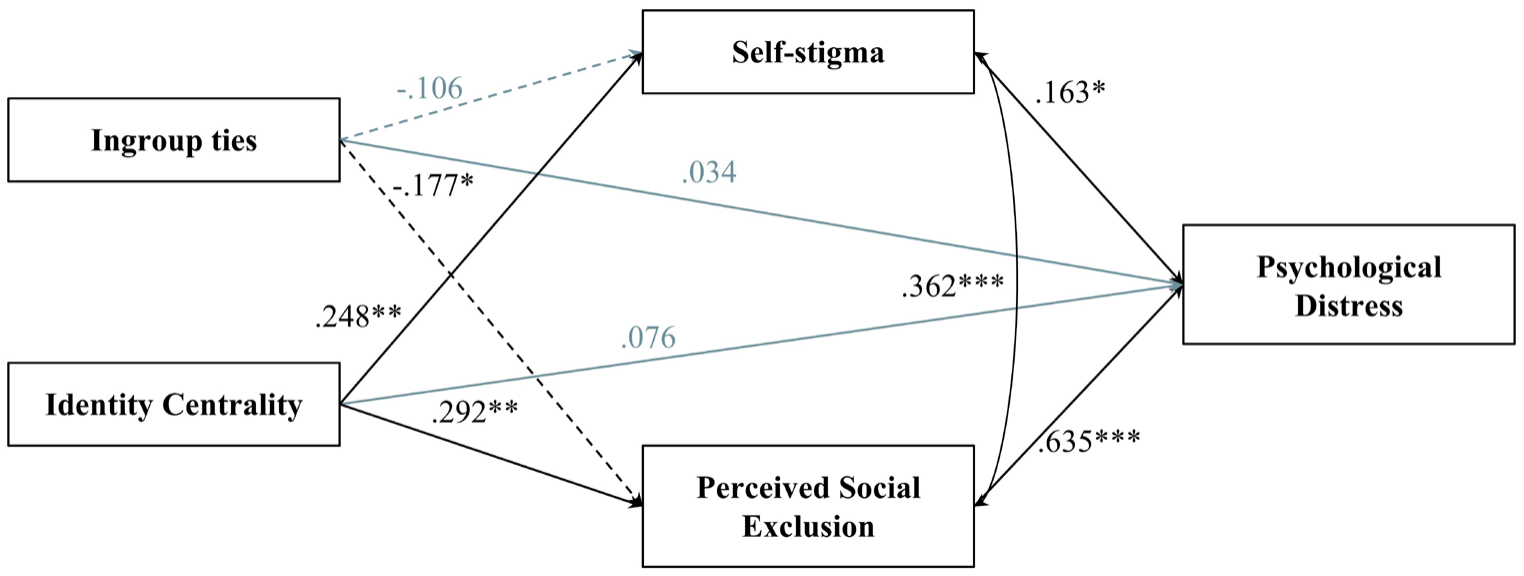
Model 1 (standardized coefficients). All direct effects are shown, whether they are significant (black lines) or not (grey lines). Negative paths are indicated by dashed lines. Indirect effects are not shown for clarity purposes. ***p < .001; **p < .01; *p < .05.

The results partially supported the proposed model (AIC = 1200.284; BIC = 1245.543). As shown in Figure 1, ingroup ties were not significantly directly associated with self‐stigma but were significantly directly associated with lower perceived social exclusion. The indirect association between ingroup ties and psychological distress via stigma was not significant (*β* = −.017, *p* = .315, 95% CI [−.061; .011]). The indirect effect of ingroup ties on psychological distress through perceived self‐exclusion was significant and negative (*β* = −.113, *p* = .045, 95% CI [−.225; −.004]), such that higher levels of ingroup ties were associated with lower perceived social exclusion, fully mediating its negative association with psychological distress. Identity centrality was both significantly and positively associated with higher self‐stigma and perceived social exclusion. In addition, its indirect effect on psychological well‐being via self‐stigma was marginally significant, as the *p‐value* was higher than .05 but the *zero* was not included in the bootstrap‐derived confidence interval (*β* = .040, *p* = .060, 95% CI [.006; .092]), suggesting that identity centrality may be indirectly positively associated with psychological distress via self‐stigma. The indirect association between identity centrality and psychological distress through perceived social exclusion was significant and positive (*β* = .185, *p* = .002, 95% CI [.065; .306]), such that higher identity centrality was associated with increased perceived social exclusion, which in turn was associated with increased psychological distress, with perceived social exclusion accounting for this indirect association in the estimated model.

The results for the alternative model, in which self‐stigma and perceived social exclusion were specified as predictors and ingroup ties and identity centrality as mediators, are reported in the Additional Analyses file (OSF folder: https://osf.io/7a4df/overview?view_only=0666c352bb474732832db7de3976eceb). Overall, the findings did not support the hypothesised associations. Specifically, in the alternative model, self‐stigma and perceived social exclusion were not significantly related to ingroup ties, identity centrality, or psychological distress. The Additional Analyses file also includes the detailed results of the model including time already spent in prison as a covariate. Time in prison was not significantly associated with any variables, and only the direct and indirect paths between ingroup ties and well‐being via perceived social exclusion remained significant, in the same direction as the original model.

## DISCUSSION

This study contributes to the Social Identity Approach to Health by examining how different dimensions of social identification, namely identity centrality and ingroup ties, can simultaneously function as either social cure or social curse processes among prisoners. Furthermore, it extends existing literature by investigating self‐stigma and perceived social exclusion as potential underlying processes consistent with the observed association. Specifically, we examined whether the associations of ingroup ties and identity centrality with psychological distress are consistent with indirect associations via self‐stigma and perceived social exclusion. We investigated these processes in the prison context, characterized by chronic exclusion and pervasive stigma, where questions about the benefits and costs of social identification are especially salient. Results suggested that social identification has both positive and negative associations with well‐being, with ingroup ties and identity centrality showing divergent indirect effects via perceived social exclusion and, to some extent, self‐stigma. Consistent with a social cure perspective, ingroup ties were associated with lower perceived social exclusion, suggesting that experiencing peer connectedness, mutual support, and feelings of belonging among fellow prisoners may help buffer the isolating nature of imprisonment. Lower perceived exclusion accounted for the indirect association between ingroup ties and psychological distress. In contrast, identity centrality showed a different pattern: the more central the prisoner identity was to the self, the more participants reported feeling excluded and self‐stigmatized. Furthermore, higher levels of perceived social exclusion fully mediated the positive association between identity centrality and psychological distress, while the indirect path through self‐stigma was only marginally significant and should be treated as suggestive rather than conclusive evidence. Notably, ingroup ties were not associated with self‐stigma, suggesting that belongingness and stigma internalization may operate through distinct pathways.

Overall, the results support the co‐presence of social cure and social curse processes, highlighting the importance of examining their interplay rather than focusing on a single mechanism. Feelings of belonging and connectedness fostered by group identification appear protective regardless of group status, whereas the personal importance of group membership may yield divergent outcomes depending on whether the group is high‐ or low‐status. Consistently, both in the present study and in prior research on marginalized populations (Bilewicz et al., 2021, 2022), as well as among non‐marginalized individuals (Ysseldyk et al., 2018), ingroup ties were associated with enhanced well‐being, while identity centrality has been linked to greater well‐being in non‐stigmatized groups (Crane et al., 2018) but to increased risk in marginalized ones (Bilewicz et al., 2021, 2022; Marinucci et al., 2023).

### Underlying mechanisms and contextual interpretation

Perceived social exclusion appears to play a central role in the investigated processes. This finding reinforces the interpretation of imprisonment as a form of social exclusion (Traversa, Marinucci, Riva, & Pancani, 2025), suggesting that it not only defines the prison experience but also shapes prisoners' psychological outcomes. Indeed, feelings of belonging and social connectedness with fellow prisoners tend to be associated with lower perceived exclusion, whereas placing greater importance on the prisoner identity tends to be associated with higher perceived exclusion, which in turn relates to contrasting patterns of well‐being. A similar dynamic may also characterize other chronically excluded groups, where the presence of a dual pathway involving ingroup ties and identity centrality has already been explored. In particular, Marinucci et al. (2023) examined how different dimensions of social identification differently affected perceived social exclusion and, in turn, well‐being among homeless people, although the effect of ingroup ties was not significant in their study. It is possible that the specific characteristics of the prison environment, particularly the loss of previous social bonds (Schaefer et al., 2017) and the enforced cohabitation with fellow prisoners (Schliehe et al., 2022), render the relational dimension of social identification especially salient. Consistently, qualitative research on detained immigrants highlighted how a prisoner identity emerged as a source of connection, support, and mutual understanding (Kellezi et al., 2019). This pattern may also help explain why reported levels of perceived social exclusion were relatively low in our sample, although social desirability or measurement factors could also have contributed. One possibility is that, given the abundance and proximity of fellow group members, exclusion from broader societal groups may feel less salient in daily prison life. Nonetheless, despite its low mean level, perceived social exclusion still appears to play a pivotal role in explaining the observed processes. Future research should further examine these dynamics, especially the role of supportive peer connectedness and feelings of belonging among fellow group members, across other marginalized populations. Comparing institutionalized groups (e.g., prisoners or individuals in addiction rehabilitation) with non‐institutionalized ones (e.g., homeless people or migrants) could help clarify how contextual factors shape the functions of social identification.

Furthermore, results suggested a limited role of self‐stigma in explaining the observed processes, particularly given its non‐significant association with ingroup ties. As self‐stigma reflects the internalization of prejudice, stereotypes, and discrimination endorsed by the broader public (Corrigan & Watson, 2002), feelings of connectedness with the ingroup may be less directly relevant. Conversely, identity centrality, reflecting the salience and personal importance of the group identity, which in the case of stigmatized groups also entails awareness of the group's negative societal image, was significantly associated with higher self‐stigma, which in turn related to lower well‐being, consistent with the Identity Threat Model of Stigma (Major & O'Brien, 2005). Moreover, self‐stigma is a complex, multidimensional process encompassing perceived stigma, stereotype agreement, and both internalized and anticipated stigma (Moore et al., 2018), thereby calling for more nuanced hypotheses and operationalisations. Additionally, in the present study, only three items were drawn from the original 39‐item scale (nine in the short version) developed by Mak and Cheung (2010), limiting the possibility of a more detailed assessment of this construct. We argue, however, that considering self‐stigma, meaning a process or internalization and response to stigma, could offer a deeper understanding of social identity dynamics among stigmatized groups. For example, individuals may recognize being targets of discrimination and consequently identify more strongly with their ingroup (Branscombe et al., 1999), yet the degree to which they internalize stigma, and its ultimate impact on well‐being, may depend on the interplay of specific risk and protective factors (Moore et al., 2018). In this sense, exploring the distinct roles of the various dimensions of social identification, as done in the present study, can provide valuable initial insights.

### Limitations and future research

The present study has several limitations that should be acknowledged. Many of them stem from the organizational and logistical challenges of conducting research in prison settings, which influenced both the study design and data collection. In particular, difficulties in accessing prison institutions limited the number and type of facilities that could be involved, resulting in a sample composed solely of male prisoners from non‐high‐security facilities, thus reducing generalisability. Notably, women and girls represent only 6.9% of the global prison population (Fair & Walmsley, 2022), meaning that male prisoners represent the large majority; nevertheless, female prisoners remain significantly understudied and future research should extend these findings to this population. Furthermore, despite the difficulties in accessing high‐security facilities, future research should endeavour to include this population, as the stricter limitations on contact between inmates may influence social identity processes and warrant specific investigation.

These same constraints also prevented the implementation of an a priori power analysis and the recruitment of a larger sample, which would have allowed for more complex models controlling for potential confounding variables. For instance, the model including time as a covariate (see the Additional Analyses file in the OSF folder: https://osf.io/7a4df/overview?view_only=0666c352bb474732832db7de3976eceb) was likely underpowered. Finally, to keep each survey wave brief and manageable for participants, only a core set of variables was repeated across waves, while others, such as those assessing social identification, were measured in one or two waves, and only subsets of items from the original scales could be used. In particular, the identity centrality subscale showed relatively low internal consistency (*α* = .60), which may reflect measurement error and should be considered with caution when interpreting the findings, as it may have attenuated or distorted the observed effects. However, the low alpha may also partly reflect the conceptual breadth of the construct, encompassing both cognitive accessibility and subjective importance of social identity, which are not necessarily redundant. Notably, a Principal Component Analysis confirmed the unidimensional structure of the subscale, with all items showing factor loadings above .40, suggesting that the items converge on the intended construct.

Furthermore, it is important to acknowledge the cross‐sectional and correlational nature of the present study, due to the practical constraints that precluded a full longitudinal approach with the available data. Specifically, the variables of interest were assessed in only two of the three waves, and the number of participants who completed both waves was insufficient to test a full longitudinal model with adequate statistical power. This precludes ruling out alternative explanations and drawing firm conclusions about the directionality of effects. It is indeed possible that psychological distress, as well as self‐stigma and perceived social exclusion, influence prisoners' social identification, rather than the opposite. In particular, based on the extensive literature on the Rejection‐Identification Model (Branscombe et al., 1999), it is plausible that stigma and social exclusion may act as predictors influencing psychological distress through their impact on ingroup ties and identity centrality. However, in this study, we focused on self‐stigma and perceived social exclusion, internal and subjective experiences that do not fully overlap with the group‐based discrimination operationalized in the Rejection‐Identification Model. As a precaution, we tested this alternative model, but results did not support the hypothesised associations (see Supplementary Materials). Regarding the potential effect of psychological distress on identification processes, especially in the prison context, where physical and mental health are often compromised (Fahmy & Mitchell, 2022; Fazel & Baillargeon, 2011), it remains possible that individuals' psychological states influence their perceptions of the group and their level of identification. Nevertheless, extensive literature within the Social Identity Approach to Health, including longitudinal and experimental studies, supports the causal influence of social identification on well‐being through both social cure and social curse processes (see, e.g., Avanzi et al., 2021; Schury et al., 2020). Future longitudinal research is needed to test the proposed mechanisms more rigorously and clarify the direction of effects.

There are also some potential theoretical and conceptual caveats to be addressed. First, the ingroup ties dimension may reflect purely interpersonal processes, such as social connectedness and support, more than genuinely group‐based identification. However, in our study as in the broader literature, ingroup ties measures usually explicitly reference the specific group, making the group dimension salient. This suggests that these processes capture the subjective meaning of belonging to that group, rather than merely reflecting interpersonal social interactions. Future research should more directly address this potential overlap, adequately operationalizing and comparing purely interpersonal processes with group‐based ones. Second, it is also possible that the two mediators, namely self‐stigma and perceived social exclusion, partially overlap, as they are moderately correlated and conceptually related. Future research should consider alternative modelling strategies, such as latent factor models, to better account for their shared variance. However, their covariance was accounted for in the estimated model, and keeping the two constructs separate allowed for a more nuanced investigation of the described processes. Indeed, self‐stigma reflected an internalized evaluative process, while perceived social exclusion captured a sense of relational deprivation.

### Theoretical and practical implications

The present study successfully applied the Social Identity Approach to Health, integrating it with research on social exclusion and stigma, to a unique and challenging context. This underscores how focusing on hard‐to‐reach populations such as prisoners can, despite their specificity, yield meaningful insights into broader psychosocial processes, such as the potential dual effect of social identification on well‐being. Beyond its theoretical relevance, this study also offered some suggestions on risk and protective factors influencing prisoners' well‐being, which is profoundly affected by the prison experience (Aldridge et al., 2018; Bedaso et al., 2020; Stoliker et al., 2021). Such insights can inform practices and policies designed to foster prisoners' positive adjustment and support rehabilitation. Specifically, strengthening positive intragroup relationships that foster a sense of belonging and connectedness, thus enhancing the ingroup ties dimension of social identification, may help promote the social cure pathway. For example, the theory‐based and evidence‐supported *Groups 4 Health* program, grounded in the Social Identity Approach to Health, aims to reduce isolation and psychological distress by helping individuals build and maintain meaningful social group connections (Haslam et al., 2016). In the prison context, previous research has shown that participation in meaningful activities can foster positive social connections, thus reducing social exclusion and promoting well‐being. For instance, a qualitative study examining prisoners' involvement in an educational programme found that such participation facilitated the formation of a prosocial peer group that provided support both during incarceration and after release (Pelletier & Evans, 2019). Likewise, Aureli et al. (2020) showed that being part of a support group in prison served as a protective factor for well‐being by increasing prisoners' perceptions of social support. In parallel, individual‐level interventions or therapeutic work could help marginalized individuals manage and resist the internalization of stigma, thereby mitigating the negative effects of a spoiled identity and preventing social curse trajectories (Pantelic et al., 2019). In the prison context, educational programmes may again play a beneficial role, as they have been described as a means of challenging the stigmatized identity associated with imprisonment and reducing the impact of self‐stigma on ex‐prisoners' self‐concept (Evans et al., 2018). More broadly, addressing the social stigma and discrimination surrounding marginalized identities such as prisoners requires action across multiple levels: interpersonal, collective, and institutional. This includes strategies centred on education and intergroup contact, as well as governmental efforts that support individual and collective initiatives aimed at reducing stigma (Heijnders & Van Der Meij, 2006).

## CONCLUSIONS

By integrating the Social Identity Approach to Health with perspectives on stigma and social exclusion, this study suggested that social identification among male prisoners may simultaneously protect and threaten well‐being, depending on the role of ingroup ties and identity centrality. More specifically, ingroup ties, which are related to experiences of peer connectedness, social support and belonging, were associated with lower perceived social exclusion and, in turn, lower psychological distress. In contrast, identity centrality, which reflects the embrace of the stigmatized prisoner identity as central to the self, was associated with higher perceived exclusion and self‐stigma and, in turn, higher distress. Beyond its specific findings, the present study shows how established psychosocial frameworks can, and should, be applied to explore social dynamics within hard‐to‐reach and marginalized populations. Such approaches provide valuable tools for interpreting complex processes, advancing theoretical understanding, and generating practical insights. In particular, they may help identify risk and protective factors for well‐being among individuals facing extreme life circumstances, such as imprisonment. Psychosocial research thus plays a crucial role not only in explaining social phenomena but also in informing policy and practice. Accordingly, this study serves as a call to extend and adapt existing psychosocial models to extreme and understudied contexts, where doing so holds both theoretical and applied relevance.

## AUTHOR CONTRIBUTIONS

**Teresa Traversa:** Conceptualization; investigation; writing – original draft; methodology; formal analysis. **Aoife‐Marie Foran:** Writing – review and editing; conceptualization. **Marco Marinucci:** Methodology; writing – review and editing. **Luca Pancani:** Methodology; writing – review and editing; supervision. **Paolo Riva:** Methodology; writing – review and editing. **Jolanda Jetten:** Conceptualization; writing – review and editing; supervision.

## CONFLICT OF INTEREST STATEMENT

The authors declare that there is no conflict of interest.

## Data Availability

The analytic code, dataset, and materials are available at this anonymized link in the OSF platform: https://osf.io/7a4df/overview?view_only=0666c352bb474732832db7de3976eceb.
